# Identification and Epidemiological Analysis of Antibiotic-Resistant Bacteria in the Oral Microbiome of the Population in Pakistan

**DOI:** 10.7759/cureus.70666

**Published:** 2024-10-01

**Authors:** Javeria Zaheer, Muhammad Naeem Khan, Atiq Ur Rahman, Muhammad Asif Shahzad, Zenab Yaasir, Madeeha Lateef, Nida Gujar

**Affiliations:** 1 Department of Microbiology, Akhtar Saeed Medical and Dental College, Lahore, PAK; 2 Department of ENT, Gomal Medical College, Dera Ismail Khan, PAK; 3 Department of Maxillofacial Surgery, Gomal Medical College, Dera Ismail Khan, PAK; 4 Department of Oral and Maxillofacial Surgery, Azra Naheed Dental College, The Superior University, Lahore, PAK; 5 Department of Dental Materials, Akhtar Saeed Medical and Dental College, Lahore, PAK; 6 Department of Biochemistry, Sardar Begum Dental College, Gandhara University, Peshawar, PAK; 7 Department of Internal Medicine, Punjab Medical College, Allied Hospital, Faisalabad, PAK

**Keywords:** antibiotic resistance, epidemiology, oral microbiome, pakistan, penicillin, tetracycline

## Abstract

Background

Antibiotic resistance in the oral microbiome poses serious health risks worldwide, particularly in developing countries like Pakistan. Public health efforts are challenged by the potential of the oral cavity to serve as a reservoir for resistant bacteria due to its frequent exposure to antibiotics.

Objective

This study aimed to identify and analyze the prevalence and epidemiology of antibiotic-resistant bacteria within the oral microbiome of the Pakistani population.

Methodology

A cross-sectional study was conducted at Akhtar Saeed Medical and Dental College, Lahore, and Gomal Medical College, Dera Ismail Khan, from January 2023 to December 2023. A total of 290 participants, aged 18 years or older, were recruited based on specific inclusion and exclusion criteria. Oral swabs were collected and analyzed using conventional culture methods. All descriptive and inferential statistical analyses were performed using SPSS version 25 (IBM Corp., Armonk, NY), with a significance level set at p <0.05.

Results

The most common antibiotic-resistant bacteria identified were *Enterococcus faecalis* (24.48%, n = 71), *Staphylococcus aureus* (27.24%, n = 79), and *Streptococcus mutans* (35.86%, n = 104). The most frequent resistances were to penicillin (32.14%, n = 93), tetracycline (23.45%, n = 68), and erythromycin (22.07%, n = 64). Recent antibiotic use was significantly associated with higher rates of resistance (p = 0.01), with 75.19% of individuals (n = 97) who had used antibiotics within the past three to six months showing resistance.

Conclusion

The study reveals a high prevalence of antibiotic-resistant bacteria, particularly to penicillin and tetracycline, in the oral microbiome of the Pakistani population.

## Introduction

Antibiotic resistance is a growing worldwide health issue that affects public health significantly, especially in underdeveloped countries [1,2]. The oral microbiome, a complex ecosystem made up of many bacterial species that might serve as a reservoir for bacteria resistant to antibiotics, is one important area of concern [3]. The oral cavity is especially susceptible to the emergence and spread of resistant bacterial strains due to its continuous exposure to external environments and the use of antibiotics for dental and medical disorders [4].

Like many other underdeveloped countries, Pakistan struggles to control antibiotic resistance because of factors such as the availability of over-the-counter antibiotics, inappropriate prescribing practices, and low public understanding of the safe use of these drugs [5]. However, Pakistan faces several challenges that are distinct from other developing nations due to its unique socioeconomic, cultural, and healthcare landscape. One significant challenge is the widespread availability of antibiotics without a prescription, a practice that is particularly rampant in Pakistan, where regulations on the sale of antibiotics are often weakly enforced [6]. This is compounded by the cultural tendency to self-medicate, which results in the misuse and overuse of antibiotics among the general public.

In addition, Pakistan's healthcare infrastructure, particularly in rural and underdeveloped areas, lacks the necessary resources to implement effective antibiotic stewardship programs. This lack of infrastructure and poor access to qualified healthcare professionals often lead to patients relying on informal medical practitioners or unregulated pharmacies, which contributes further to the misuse of antibiotics [7]. Unlike in some other developing countries, where there may be government-led initiatives or international aid programs targeting antibiotic use, Pakistan’s healthcare system faces significant internal hurdles, including political instability, that hamper the development and enforcement of such measures.

A unique aspect of the Pakistani context is the high prevalence of counterfeit or substandard antibiotics in the pharmaceutical market. This issue exacerbates antibiotic resistance because low-quality drugs may not effectively eliminate bacterial infections, leading to prolonged exposure and greater opportunities for resistance to develop [8]. The oral microbiome's resistance to antibiotics has mostly been the subject of research conducted in industrialized nations; information from developing areas such as Pakistan is scarce [9,10]. Studies conducted in Pakistan, including one with 271 participants showing a 94% prevalence of oral health impact (with physical pain and psychological disability as the most affected domains), highlight the need for further research into oral health-related quality of life and clinical conditions like caries [9].

Despite a study finding no significant association between diabetes status and periodontal disease severity [10], these findings suggest that other risk factors, such as poor oral health behaviors and psychological distress, may play a more significant role, underscoring the urgent need for comprehensive epidemiological studies and targeted interventions in the region. This practice, combined with improper waste disposal and water contamination, allows resistant bacteria to circulate between humans, animals, and the environment, creating a complex and uncontrolled spread of antibiotic resistance [11]. While other developing countries also face issues with agricultural antibiotic use, the lack of regulation and enforcement in Pakistan creates a particularly severe challenge in managing resistance in this sector.

The oral health sector in Pakistan is significantly impacted by these issues due to the high frequency of dental infections and periodontal illnesses that require regular antibiotic usage [12]. Poor access to dental care and the use of antibiotics as a substitute for proper dental treatments, especially in underprivileged areas, further contribute to the problem. This reliance on antibiotics rather than preventive or curative dental care accelerates the emergence of antibiotic-resistant bacteria in the oral microbiome [13]. The cultural norms surrounding oral health, where dental check-ups are often neglected until problems become severe, increase the likelihood of antibiotic misuse as patients seek quick fixes for long-standing dental issues.

In contrast to many industrialized nations where extensive research has been conducted on antibiotic resistance in the oral microbiome, data from Pakistan and other developing regions remain scarce [7-9,14]. The lack of large-scale studies hampers efforts to understand the full scope of the problem, and the few studies that have been conducted are often limited in focus, examining only certain bacterial species or small population samples. This lack of comprehensive data makes it difficult to develop targeted strategies to combat antibiotic resistance in these regions [15].

This study addresses the research gap by offering a detailed identification and epidemiological analysis of antibiotic-resistant bacteria in the oral microbiome of the Pakistani population. By examining the prevalence and patterns of antibiotic resistance within this specific demographic, our research provides valuable insights into the public health implications and informs future interventions aimed at mitigating resistance in oral bacteria. Understanding these patterns in Pakistan’s unique context will contribute to the global effort to combat antibiotic resistance, particularly in under-researched and high-risk areas like the oral microbiome in developing nations.

Research objective

The study objective was to identify and analyze the prevalence and epidemiology of antibiotic-resistant bacteria within the oral microbiome of the Pakistani population.

## Materials and methods

Study design and setting

A cross-sectional study was conducted at Akhtar Saeed Medical and Dental College, Lahore, and Gomal Medical College, Dera Ismail Khan, along with their affiliated hospitals, Akhtar Saeed Trust Hospital and Mufti Mehmood Memorial Teaching Hospital. The research took place between January 2023 and December 2023. The primary aim of this study was to determine the prevalence and patterns of antibiotic-resistant bacteria in the oral microbiome of the Pakistani population.

Sampling method

Convenience sampling was used for recruiting participants due to its practicality in hospital and community settings. Participants were selected based on their accessibility and willingness to participate. This non-probability sampling method allowed for a more straightforward recruitment process in real-world settings. Participants were identified as recent antibiotic users through self-reported histories of antibiotic use in the past 12 months, which were confirmed using a structured questionnaire.

Inclusion and exclusion criteria

Eligible participants were aged 18 and above, with a history of antibiotic use in the past 12 months, and provided written informed consent. The exclusion criteria included individuals with chronic systemic illnesses, pregnant or lactating women, and those who had undergone any dental procedures or treatments within the previous 30 days. The 30-day exclusion period was established to prevent the influence of recent treatments on antibiotic resistance patterns.

Sample size

The study initially recruited 296 participants using a convenience sampling method. However, six participants were excluded due to not meeting the inclusion criteria (such as recent dental procedures or incomplete data). Thus, the final sample size comprised 290 participants. 

Data collection

Oral swabs were collected from the gingival margins, buccal mucosa, and dorsum of the tongue using sterilized cotton swabs. Samples were placed in a commercially available transport medium Amies agar swab medium immediately after collection to maintain the viability of the bacterial specimens. Swabs were then transported to the microbiology lab for processing.

Samples were cultured using standard bacteriological techniques on selective and differential media to isolate bacterial strains. Gram staining was performed to differentiate bacterial species, and biochemical tests were conducted for further identification. Antibiotic susceptibility testing was done using the Kirby-Bauer disk diffusion method to assess resistance against a panel of commonly prescribed antibiotics.

Structured questionnaires with closed-ended questions were administered to all participants by trained research assistants to minimize reporting bias. These questionnaires collected information on demographics, medical history, and antibiotic usage. To reduce selection bias, all data collection and sample processing followed a standardized protocol. Additionally, double data entry was employed to limit input errors.

Statistical analysis

Data were analyzed using SPSS version 25 (IBM Corp., Armonk, NY). Descriptive statistics were used to summarize demographic (age, gender) and clinical characteristics (medical, antibiotic, and dental procedure history). Chi-square tests were applied to investigate the association between antibiotic usage and the occurrence of antibiotic-resistant bacteria. Statistical significance was set at p <0.05.

Ethical statement

The study protocol was approved by the Ethical Review Committees of Akhtar Saeed Medical and Dental College and Gomal Medical College. All participants provided written informed consent before data and sample collection. The study followed the ethical principles outlined in the Declaration of Helsinki, ensuring that participants' privacy was maintained and data confidentiality was strictly observed.

## Results

The demographic details of the 290 participants are shown in Table 1. The bulk (n = 96, 33.10%) were in the 26-35 age range, followed by those in the 18-25 age range (n = 84, 29.31%). There were 128 women (44.83%) and 162 men (55.17%) in the gender distribution. Among the participants, 127 (43.79%) belonged to the middle class, 121 (41.72%) to the lower class, and 42 (14.48%) to the upper class. The participants' educational backgrounds ranged as follows: 124 (42.76%) had a university degree, 81 (27.93%) had finished college, 61 (21.03%) had just completed elementary school, and 24 (8.28%) had no formal education at all. In terms of employment, 147 individuals (50.69%) were working, 53 (18.28%) were in school, 42 (14.48%) were jobless, 28 (9.66%) were retired, and 20 (6.90%) were classified as other.

**Table 1:** Demographic Characteristics of Participants (n = 290) *Chi-square test was applied and p-value <0.05 was taken significant.

Characteristic		Number of Patients (n = 290)	Percentage	Resistance Rate (%)	p-Value*
Age (years)	18-25	84	29.31	42.86	0.02
	26-35	96	33.10	78.13	0.01
	36-45	53	18.28	71.7	0.03
	46-55	37	12.76	56.76	0.12
	56-65	13	4.48	61.54	0.15
	Above 65	7	2.41	71.43	0.08
Gender	Male	162	55.17	74.07	0.03
	Female	128	44.83	49.22	0.05
Socioeconomic Status	Low	121	41.72	61.98	0.04
	Middle	127	43.79	67.72	0.02
	High	42	14.48	52.38	0.1
Education Level	No Formal Education	24	8.28	58.33	0.06
	School	61	21.03	49.18	0.08
	College	81	27.93	49.38	0.07
	University	124	42.76	79.84	0.01
Occupation	Student	53	18.28	49.06	0.1
	Employed	147	50.69	74.83	0.04
	Unemployed	42	14.48	61.9	0.09
	Retired	28	9.66	46.43	0.11
	Other	20	6.90	40	0.15

**Figure 1:**
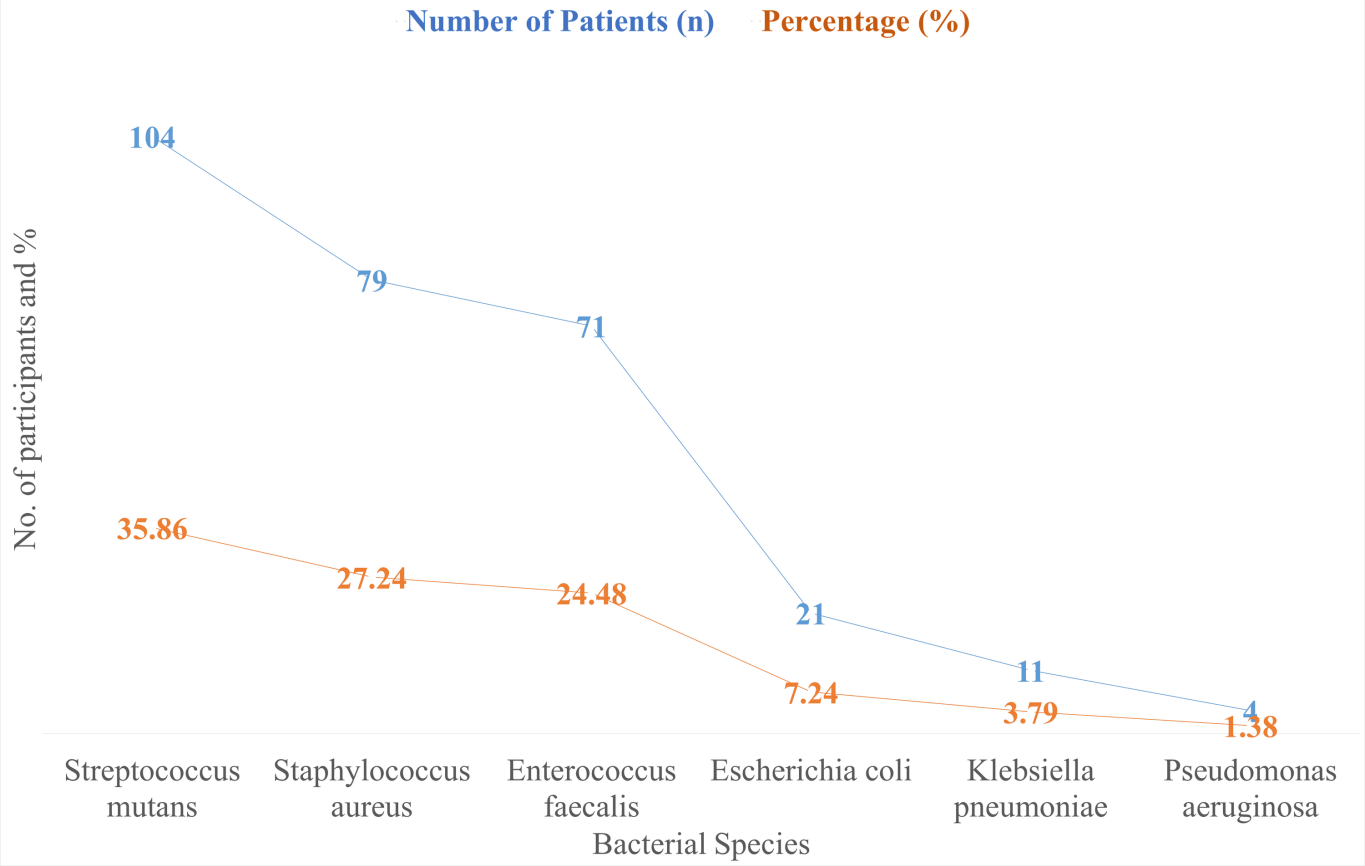
Prevalence of Antibiotic-Resistant Bacteria (n = 290)

The frequency of microorganisms resistant to antibiotics in 290 patients is shown in Figure 1. The most common species, *Streptococcus mutans*, was found in 104 individuals and accounted for 35.86% of cases. Seventy-one patients (24.48%) had *Enterococcus faecalis* and 79 patients (27.24%) had *Staphylococcus aureus*. Eleven patients (3.79%) had *Klebsiella pneumoniae* and 21 patients (7.24%) had *Escherichia coli*. Four individuals (1.38%) had *Pseudomonas aeruginosa*.

**Figure 2:**
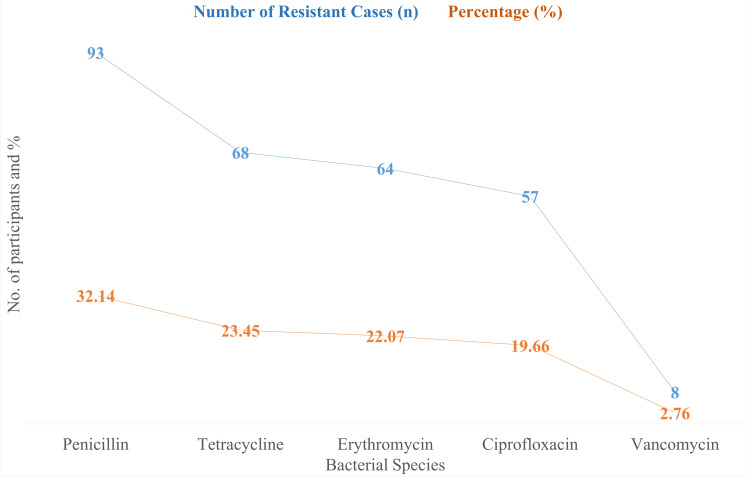
Antibiotic Resistance Patterns (n = 290)

The patterns of antibiotic resistance among 290 patients are shown in Figure 2. The most often resisted antibiotic was penicillin, which was present in 93 individuals (32.14%). Of the instances, 68 individuals had tetracycline resistance, or 23.45% of the cases. A total of 64 patients, or 22.07%, had erythromycin resistance. Ciprofloxacin resistance was observed in 57 cases, or 19.66% of the total. Only eight patients, or 2.76% of the sample as a whole, were affected by vancomycin, which had the lowest resistance rate.

Table 2 illustrates the association between the duration of antibiotic use and the prevalence of antibiotic-resistant strains among the 290 participants. Among those who used antibiotics within the 1-3-month period (n = 129), a significant 75.19% (n = 97) exhibited antibiotic resistance. In the 4-6 months category (n = 40), 52.50% (n = 21) displayed resistance, while 44.44% (n = 12) showed resistance among participants who had used antibiotics within 7-12 months (n = 27). Notably, in the Duration Not Confirmed group (n = 94), 56.38% (n = 53) of participants also exhibited antibiotic resistance. The results reveal a statistically significant association (p = 0.01) between recent antibiotic usage and the development of resistance, highlighting that a greater percentage of individuals who used antibiotics recently exhibited resistant strains compared to those with less clearly defined usage durations.

**Table 2:** Association Between Duration of Antibiotic Use and Development of Resistant Strains *Chi-square test (χ² test) of independence.

Time Since Last Antibiotic Use	Total Number of Patients	Percentage of Total Patients	Number of Patients With Resistant Strains	Percentage of Patients With Resistant Strains (%)	Percentage of Patients Exhibiting Resistance Based on Total (%)	p-Value*
Duration Not Confirmed	94	32.41	53	56.38	18.28	0.01
1-3 Months	129	44.48	97	75.19	33.45	
4-6 Months	40	13.79	21	52.5	7.24	
7-12 Months	27	9.31	12	44.44	4.14	
Total	290	100	183	63.1	63.1	

## Discussion

The present research offers a thorough examination of antibiotic-resistant bacteria in a population's oral microbiome from Pakistan, providing important new information on the frequency and antibiotic resistance patterns of these pathogens. The most common antibiotic-resistant bacterium, according to the data, is *Streptococcus mutans*, which was found in 35.86% (n = 104) of the individuals. *Enterococcus faecalis* (24.48%, n = 71) and* Staphylococcus aureus* (27.24%, n = 79) were the next most common bacteria. This distribution is consistent with results from previous studies carried out in comparable environments [14]. For example, while *Streptococcus mutans* has a little lower incidence, an Indian research found that it is one of the most resistant strains in the oral microbiome [15].

According to the findings of this research, 32.14% (n = 93) of the participants had penicillin-resistant bacterial strains, while 23.45% (n = 68) had tetracycline-resistant bacterial strains. These results align with worldwide trends, which indicate that oral bacteria commonly exhibit antibiotic resistance to both tetracycline and penicillin [16]. The reduced incidence of vancomycin-resistant bacterial strains (2.76%, n = 8), however, contrasts with international studies that have reported higher rates of antibiotic resistance [17].

Among the people who had used antibiotics in the previous three to six months, 75.19% (n = 97) had antibiotic-resistant bacteria, indicating a strong association between recent antibiotic usage and antibiotic-resistant bacteria. This result corroborates studies of a similar kind carried out in other developing nations, where the use of antibiotics in the recent past has been substantially linked to higher rates of antibiotic-resistant bacteria [18]. Notably, the research discovered that 56.38% (n = 53) of individuals who had not used antibiotics recently showed antibiotic-resistant bacteria, indicating the possibility of residual antibiotic resistance developing. This finding is consistent with other research showing that the oral microbiota may develop antibiotic resistance even in the face of little or no antibiotic exposure [19].

According to the demographic study, antibiotic resistance was seen in people of all ages, with the age group of 26-35 having the greatest prevalence (33.10%, n = 96). This is in line with recent research that has shown the possibility of widespread antibiotic resistance in adults, especially in those who take antibiotics frequently [20]. Our study aligns with Ortega-Paredes et al. [21] and Mostafa et al. [22], showing education's influence on antibiotic knowledge, with 42.76% of our participants having a university education, 64.88% correct responses in the Ecuadorian study, and 35.1% of Egyptian students having sufficient health literacy. However, misconceptions persist, such as linking antibiotics to viral illnesses, with 92.2% of Egyptian students showing limited awareness of antibiotic resistance. 

The findings of this study underscore the urgent need for improved public health strategies and antibiotic stewardship, particularly in Pakistan, where resistance to penicillin and tetracycline is prevalent. To address these challenges, several initiatives can be proposed. First, educational campaigns aimed at both healthcare providers and the general public are necessary to reduce the inappropriate use of antibiotics. Health literacy programs can significantly enhance antibiotic awareness, particularly among non-medical populations. Introducing antibiotic stewardship programs in dental practices could guide appropriate prescription practices, which is crucial given the high levels of antibiotic resistance observed in *Streptococcus mutans* and *Staphylococcus aureus*. Further, public health campaigns focusing on the risks of antibiotic resistance within the oral microbiome could promote more informed decision-making and reduce self-medication practices.

Study strengths and limitations

This study has several strengths. First, it was conducted across two distinct medical institutions and their affiliated hospitals, providing a broader representation of the Pakistani population than studies conducted at a single site. Additionally, the use of standardized protocols for sample collection, transport, and processing, alongside structured questionnaires administered by trained assistants, minimized reporting and selection biases, ensuring the reliability of data. The study also employed the Kirby-Bauer disk diffusion method for antibiotic susceptibility testing, which is a well-established and reliable technique for assessing bacterial antibiotic resistance patterns.

This research project has also a couple of limitations. The cross-sectional design restricts our ability to establish causality between antibiotic use and antibiotic resistance patterns. Additionally, convenience sampling, while practical for recruitment, may not be fully representative of the general population, potentially limiting the generalizability of the results. Future studies should adopt longitudinal approaches and multicenter designs to better track changes over time and validate the findings in a broader context.

## Conclusions

The oral microbiome of the Pakistani population contains a notable amount of antibiotic-resistant bacteria, namely *Streptococcus mutans*, *Staphylococcus aureus*, and *Enterococcus faecalis*, according to this research. There is an urgent need for better antibiotic stewardship due to the high rates of penicillin and tetracycline resistance, particularly among recent antibiotic users. The existence of resistance in people who haven't used antibiotics recently points to a larger problem with residual resistance. These results highlight the need for improved monitoring and public health initiatives to successfully tackle antibiotic resistance in developing nations such as Pakistan.
